# Study of Wrist Joint Movements, Hand Dimensions, and Comparison to Grip Strength Among Omani Medical Students: A Pilot Study

**DOI:** 10.3390/jfmk11030356

**Published:** 2026-09-07

**Authors:** Mohammed Al Maskari, Muanis Al Khusaibi, Noura Al Harsoosi, Jouri Al Kaabi, Srijit Das

**Affiliations:** Department of Human & Clinical Anatomy, College of Medicine & Health Sciences, Sultan Qaboos University, Al Khoud, Muscat 123, Oman; s142074@student.squ.edu.om (M.A.M.); s141199@student.squ.edu.om (M.A.K.); s140397@student.squ.edu.om (N.A.H.); s136405@student.squ.edu.om (J.A.K.)

**Keywords:** grip strength, wrist joint, flexion, extension, ulnar deviation, radial deviation, hand anthropometric measurements

## Abstract

**Background**: Wrist joint position and hand anthropometric measurements may affect grip strength and hand performance. This pilot, analytical cross-sectional study aimed to investigate the relationship between different wrist positions and grip strength, as well as the association between grip strength and hand size in healthy individuals. **Methods**: An analytic cross-sectional study was conducted with undergraduate medical students. We assessed grip strength using a hand dynamometer in different wrist positions, including wrist flexion, extension, ulnar deviation, and radial deviation. Hand anthropometric measurements, including HB, MHB, PL, TFL, IFL, MFL, RFL, and LFL, were performed using a sliding digital caliper. **Results**: Grip strength varied significantly across wrist positions, with the highest mean value observed in the neutral position (30.53 kg), followed by ulnar deviation (28.94 kg), radial deviation (18.02 kg), extension (14.75 kg), and flexion (14.67 kg). The repeated-measures ANOVA demonstrated significant main effects of wrist position and sex, as well as a significant interaction between wrist position and sex (all *p* < 0.001). Males demonstrated higher mean grip strength than females in the neutral position (42.56 ± 8.98 kg vs. 18.54 ± 4.20 kg, respectively). Grip strength measurements across wrist positions showed strong positive correlations, with the strongest associations observed between flexion and neutral grip strength (ρ = 0.84) and between ulnar deviation and neutral grip strength (ρ = 0.81). The wrist angle was negatively correlated with grip strength during flexion (ρ = −0.24, *p* < 0.05) and extension (ρ = −0.41, *p* < 0.05), representing weak and moderate associations, respectively. Females demonstrated greater mean flexion and extension than males, whereas males demonstrated higher mean values for ulnar and radial deviation. Among males, grip strength was significantly positively correlated with maximum hand breadth (ρ = 0.365, *p* = 0.001), hand breadth (ρ = 0.326, *p* = 0.003), palm length (ρ = 0.304, *p* = 0.006), thumb length (ρ = 0.289, *p* = 0.009), and index finger length (ρ = 0.229, *p* = 0.039). No statistically significant associations were observed between grip strength and the assessed hand anthropometric measurements among females. **Conclusions**: Grip strength is markedly influenced by wrist position, with the highest grip strength measured in the neutral position and decreased in flexion, extension, or radial deviation. The results of this study may have implications for rehabilitation, ergonomic design, and sports performance in the future.

## 1. Introduction

Handgrip strength is an important indicator of muscle function, overall physical capability, and health status and has been proposed as a potential vital sign of health [1,2]. Reduced grip strength is associated with poorer functional performance and adverse health outcomes, making it a valuable indicator in clinical practice and research [3]. It is widely recognized as a measure of muscle strength and physical fitness and is increasingly used in sports science for talent identification, performance monitoring, and assessment of athletic performance [4].

The upper extremities are highly specialized structures that play a vital role in daily life, enabling a wide range of functional movements required for activities of daily living, such as reaching, lifting, grasping, and controlling motor functions. The coordinated actions of the shoulder, elbow, wrist, and hand joints allow the upper limb to position the hand in space and manipulate objects effectively [5].

The wrist joint (radiocarpal joint) is a complex synovial articulation that forms a functional connection between the forearm and hand. It provides the mobility and stability required for effective hand positioning and object manipulation during functional activity. The wrist permits flexion, extension, radial deviation, and ulnar deviation, whereas coordinated forearm motion contributes to pronation and supination. These movements are essential for performing activities of daily living, occupational tasks, and sports. A comprehensive understanding of wrist kinematics is important for evaluating hand function, as normal wrist motion plays a critical role in achieving functional performance and maintaining upper extremity biomechanics [6].

Measuring wrist range of motion (ROM) is a fundamental component of physical examination and plays an important role in evaluating, diagnosing, treating, and monitoring patients with wrist disorders [7]. Wrist position is an important factor in grip strength because wrist posture influences muscle length, tendon tension, and the mechanical efficiency of the forearm flexor muscles. Changing the wrist to an inappropriate position can significantly decrease the generated force [8]. Limitations in wrist ROM can be caused by wrist pain, muscle weakness, or disability, which may lead to a decrease in grip strength [9]. Therefore, accurate knowledge of the changes in grip strength in different wrist positions, especially ulnar and radial deviation, is important for improving hand performance, preventing injury, and providing guidelines for treatment programs for individuals with wrist or hand disorders.

The human hand is a highly specialized and biomechanically complex structure essential for performing activities of daily living, occupational tasks, and sports-related activities. Its function depends on the coordinated interaction of skeletal, muscular, and joint components, which enable the strength, dexterity, and precise motor control of the hand. A comprehensive understanding of hand biomechanics, including joint mobility and functional performance, is important for the assessment of hand function, identification of functional impairments, and development of effective rehabilitation strategies [4]. Furthermore, grip strength is widely recognized as an important indicator of neuromuscular strength and physical function, making it a valuable measure in clinical and research settings [10].

The main aim of the present study was to (i) investigate the relationship between wrist joint range of motion (flexion, extension, radial deviation, and ulnar deviation) and grip strength in males and females, and (ii) examine the association between hand anthropometric measurements and grip strength. Understanding these relationships may provide valuable insights into the factors influencing hand function and support the assessment and rehabilitation of wrist and hand disorders.

## 2. Materials and Methods

This analytic cross-sectional study was conducted at the College of Medicine and Health Sciences, Sultan Qaboos University (SQU), and focused on the relationship between wrist joint range of motion, hand anthropometric measurements, and grip strength. All the participants were Omani undergraduate medical students. Medical students aged 18–25 years were recruited because they were readily accessible for participation. Only students with no history of upper limb surgery and those who provided informed consent were included in the study. Participants who were not undergraduate medical students at SQU, were younger than 18 years or older than 25 years, had a history of upper limb surgery, had any vigorous muscular activity before the test, or did not provide consent were excluded from the study. All measurements were taken during students’ availability during university working hours from 8 a.m. until 4 p.m. All participants provided their consent by scanning a QR code. All subjects were briefed about the methods for the experiment for about ten minutes before the procedure.

The sample size was determined a priori based on the primary statistical analyses. For the primary analysis examining the effects of wrist position, sex, and their interaction on grip strength, a mixed-design repeated-measures ANOVA was performed. A medium effect size (Cohen’s f = 0.25) was assumed according to the conventional effect size criteria [11], with a significance level of 0.05 and 80% statistical power. The analysis included two sex groups and five repeated wrist position measurements, with an assumed correlation of 0.50 among repeated measurements and nonsphericity correction of 1.0. Using G*Power 3.1 [12], the minimum required sample size for the repeated-measures analysis was estimated to be 40 participants. For the second primary objective, which examined the association between grip strength and hand anthropometric measurements, a separate sample size calculation was performed for the Pearson correlation between maximum hand breadth (MHB) and grip strength. An anticipated correlation coefficient of r = 0.241 was adopted from a previous study that reported an association between maximum hand breadth and grip strength in healthy adults [13]. Using a two-tailed significance level of 0.05 and 80% statistical power, the minimum required sample size for detecting this association was estimated to be 133 participants. The larger of the two calculated sample size requirements was considered the minimum required sample size for the study. A total of 162 medical students, equally divided by sex (81 males and 81 females), were included in this study. Measurements were obtained from the dominant upper limb of each participant.

**Figure 1:**
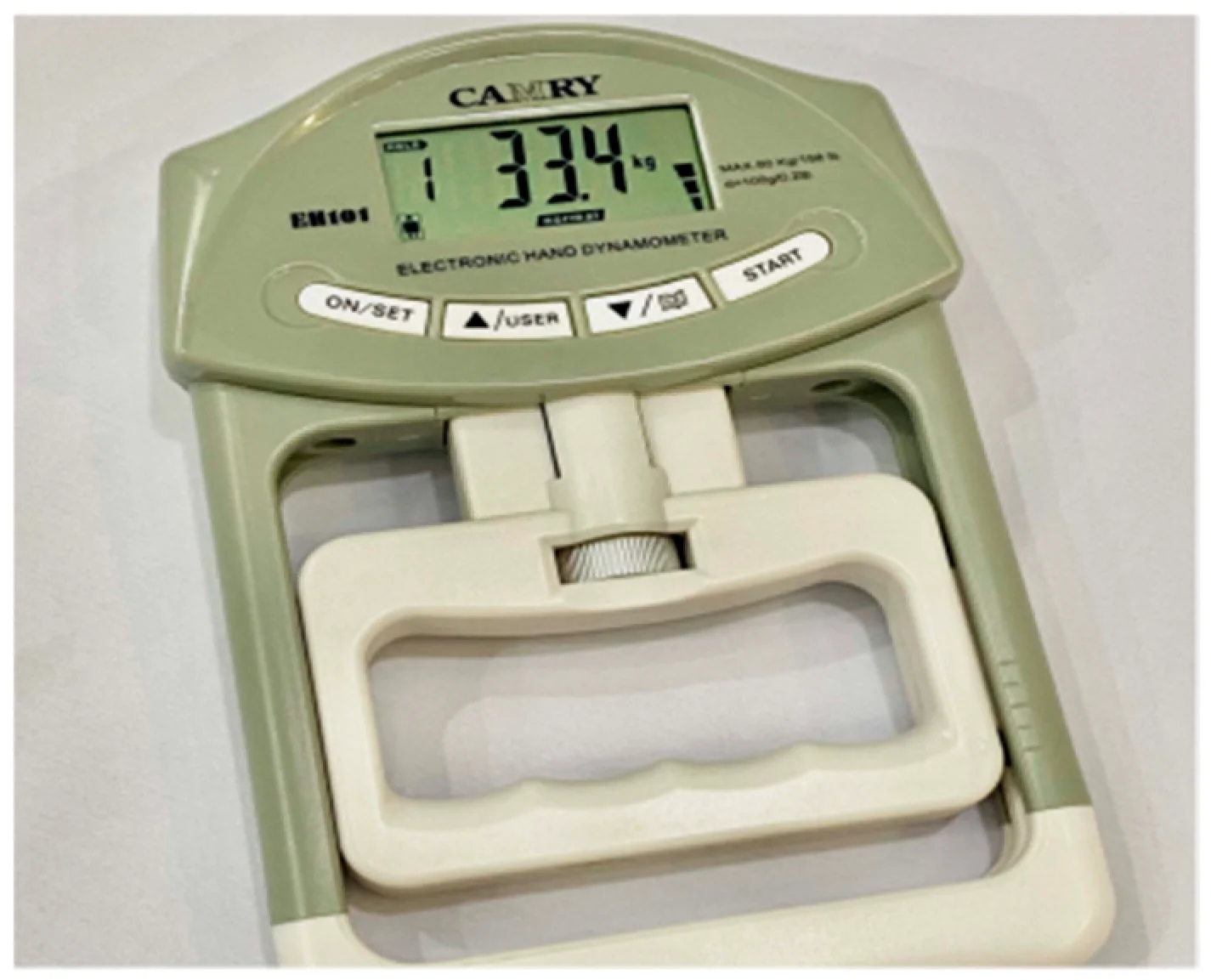
Hand dynamometer to assess grip strength [16].

Grip strength measurement was performed according to standardized testing procedures commonly recommended for clinical and epidemiological studies to ensure reliable and reproducible measurements [14]. Grip strength was measured using a CAMRY digital hand dynamometer (Figure 1), which was calibrated before each testing session. The participants were seated with their feet flat on the floor, backs upright, shoulders adducted, elbows flexed at 90°, and wrists in a neutral position. The participants were instructed to exert maximal force on the dynamometer, and the peak grip strength was recorded in kilograms (kg) [15,16]. To ensure consistency, all baseline grip strength measurements were obtained with the wrists in a neutral position.

**Figure 2:**
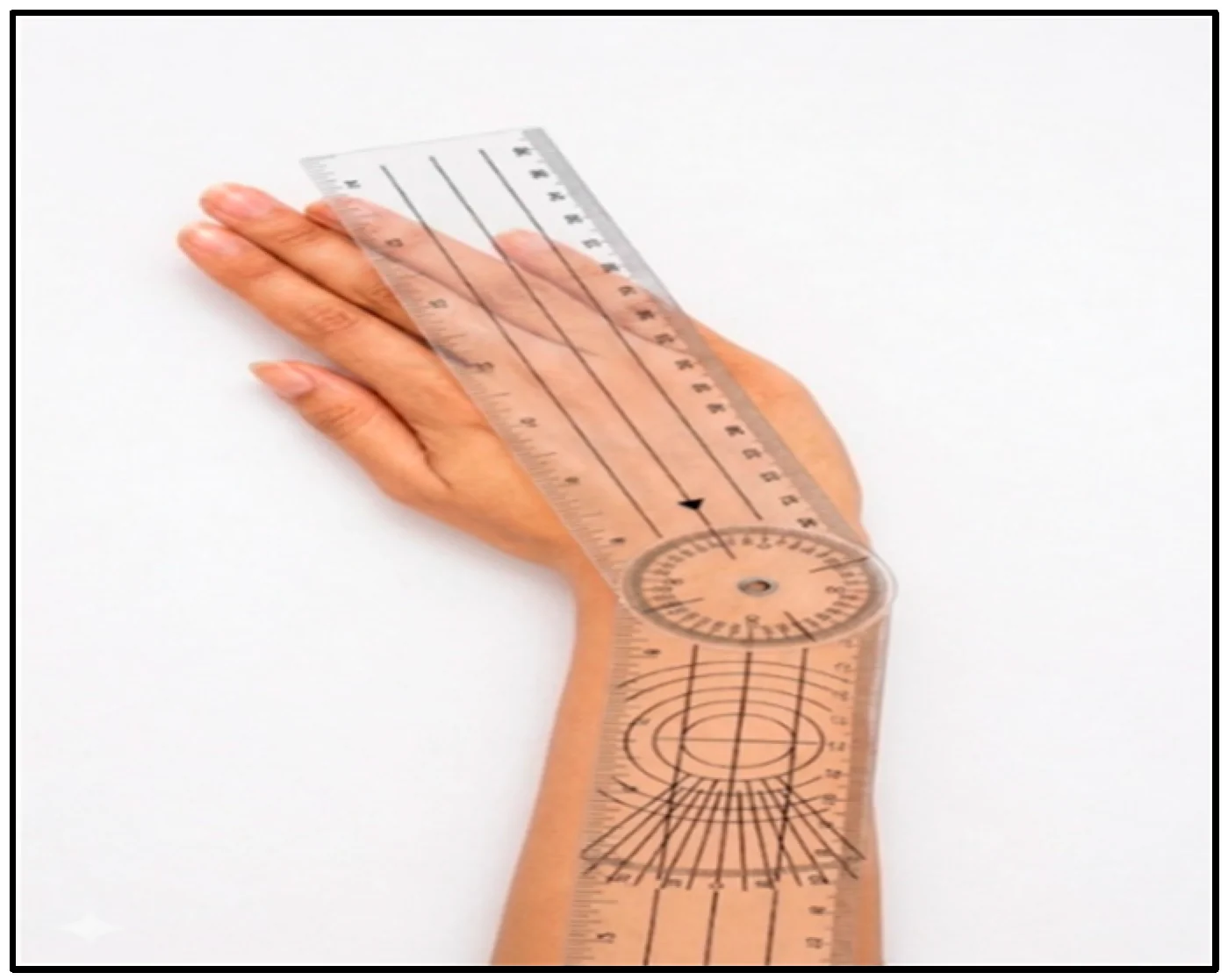
Manual goniometer used for assessing radial deviation.

**Figure 3:**
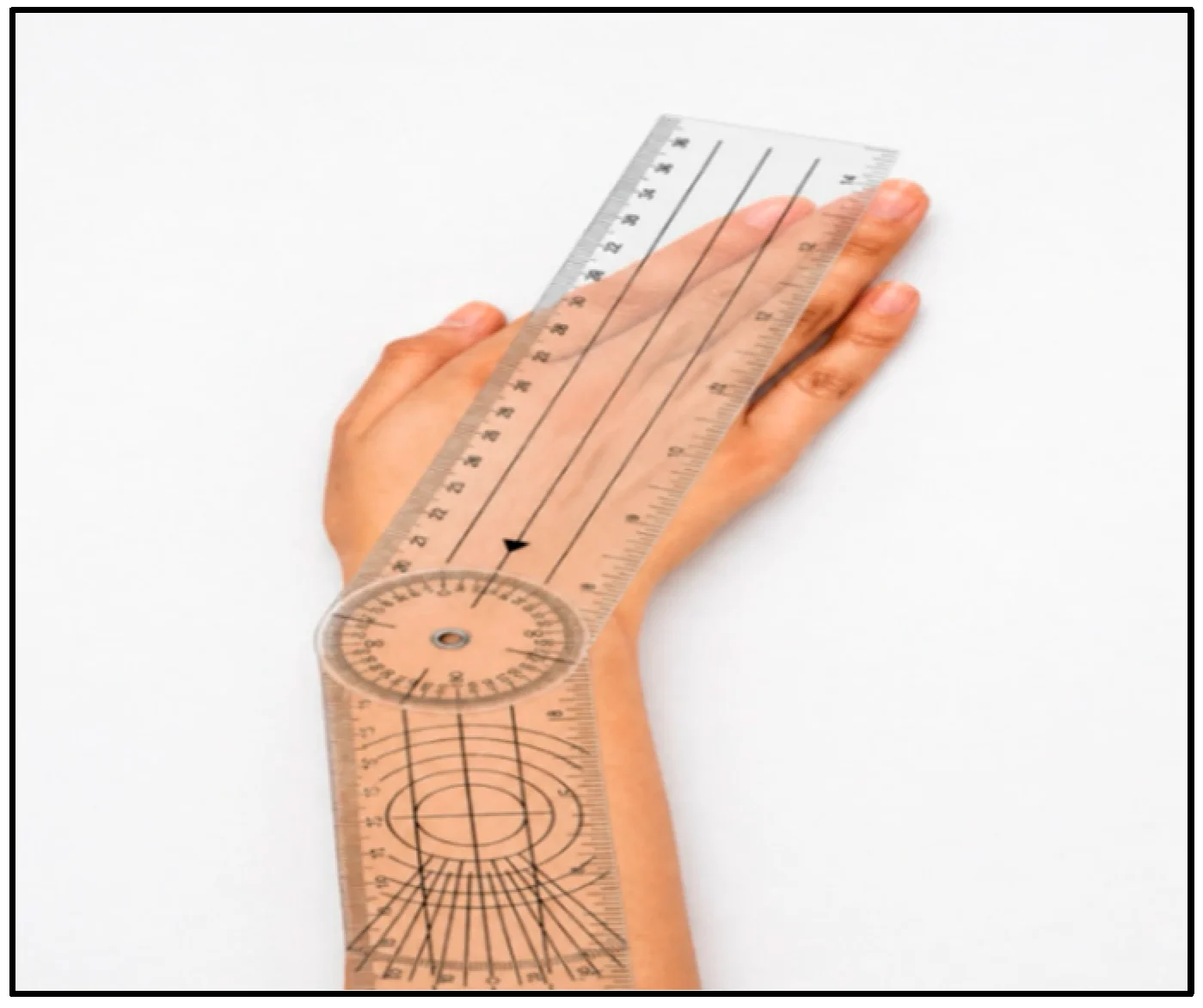
Manual goniometer used for assessing ulnar deviation.

**Figure 4:**
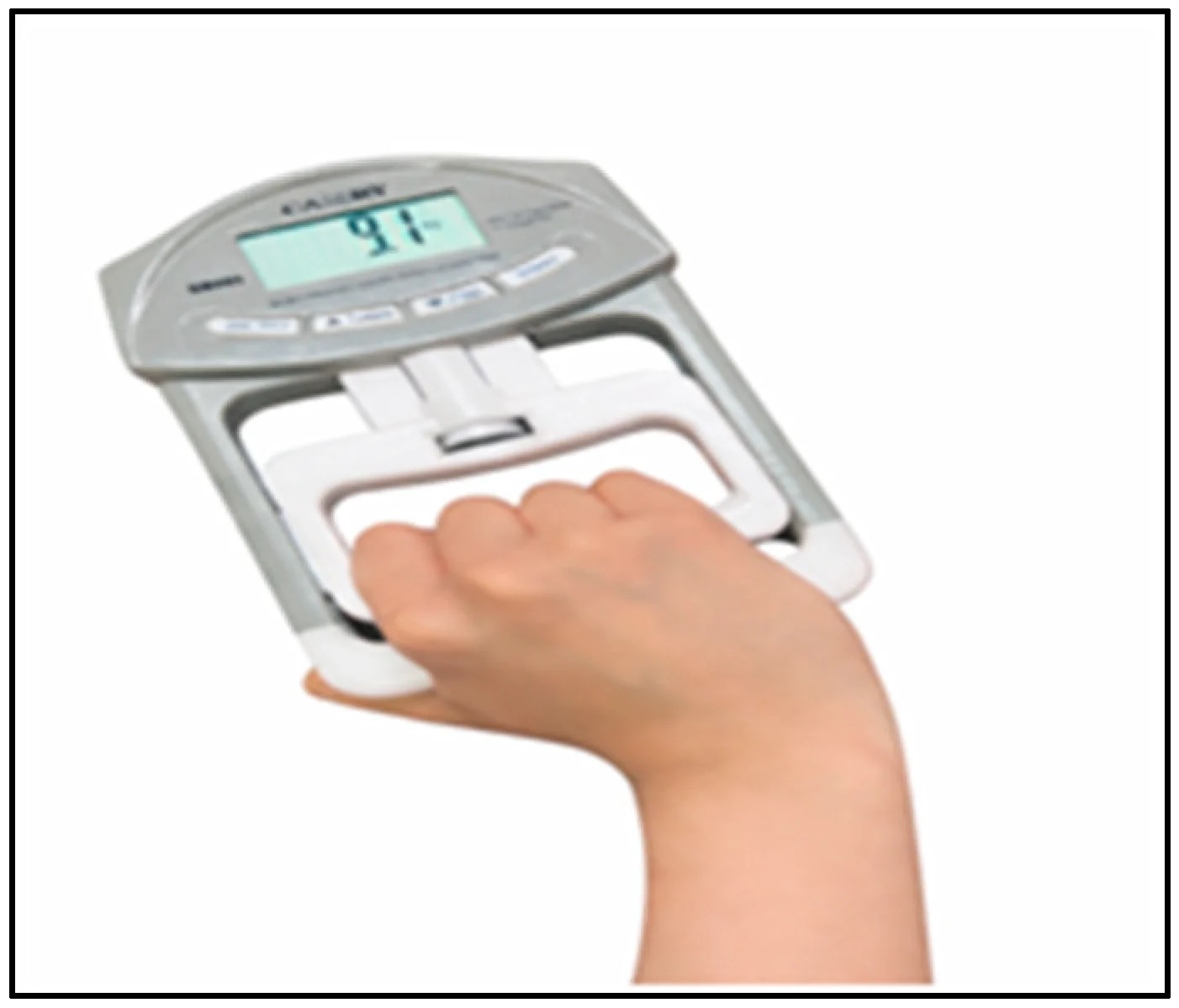
Measurement of grip strength during radial deviation.

**Figure 5:**
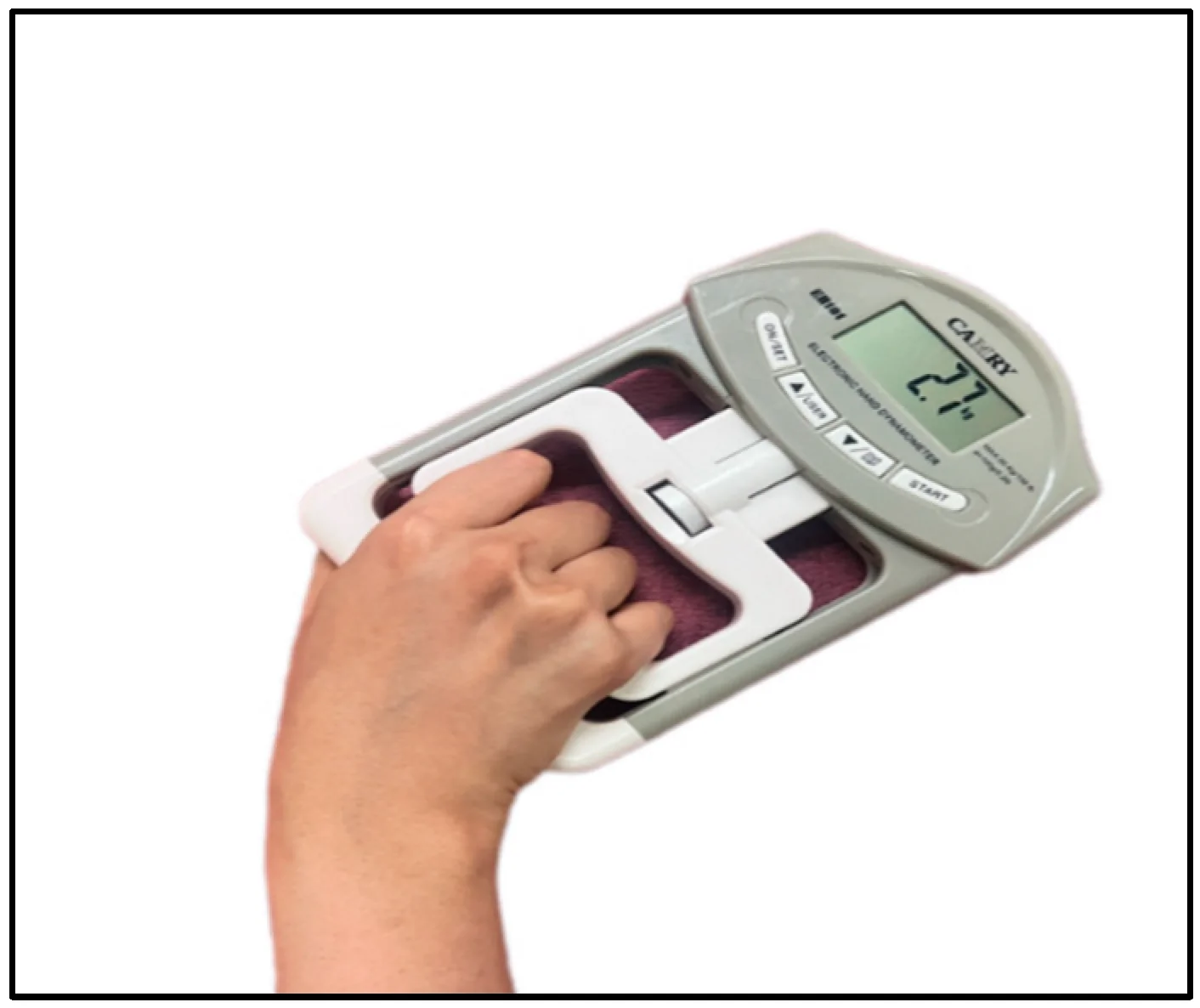
Measurement of grip strength during ulnar deviation.

The wrist ROM was assessed using a manual goniometer. We followed the protocol used in a previous study [17]. For radial and ulnar deviation measurements, the goniometer axis was positioned over the capitate bone, with the stationary arm aligned along the dorsal midline of the forearm and the moving arm aligned with the third metacarpal. The participants were instructed to actively move their wrists to the maximum radial deviation (Figure 2) and maximum ulnar deviation (Figure 3), and the corresponding angles were recorded [17]. Grip strength was subsequently measured while maintaining maximal radial deviation (Figure 4) and maximal ulnar deviation (Figure 5) using the hand dynamometer [16].

**Figure 6:**
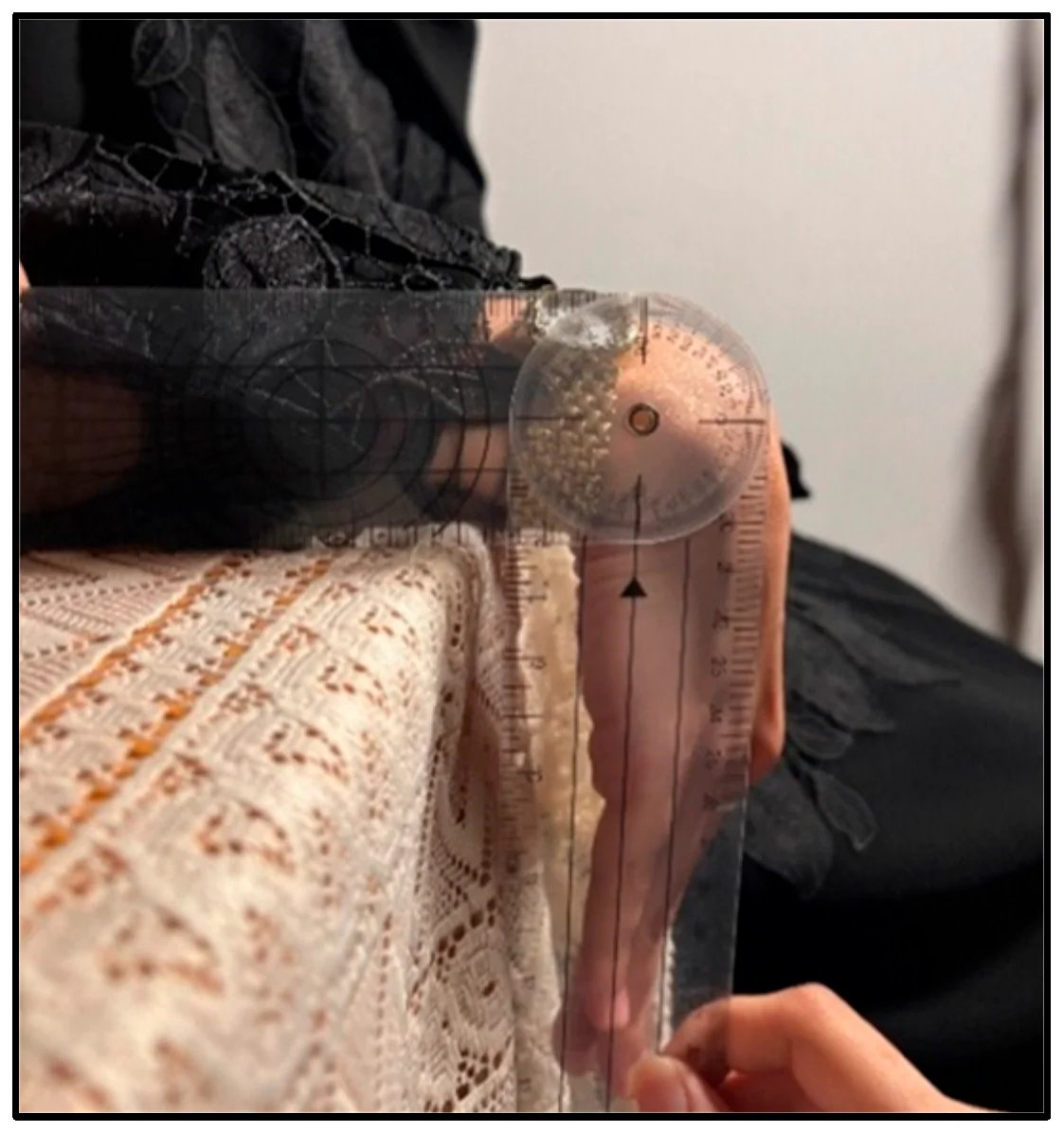
Manual goniometer used for assessing wrist flexion.

**Figure 7:**
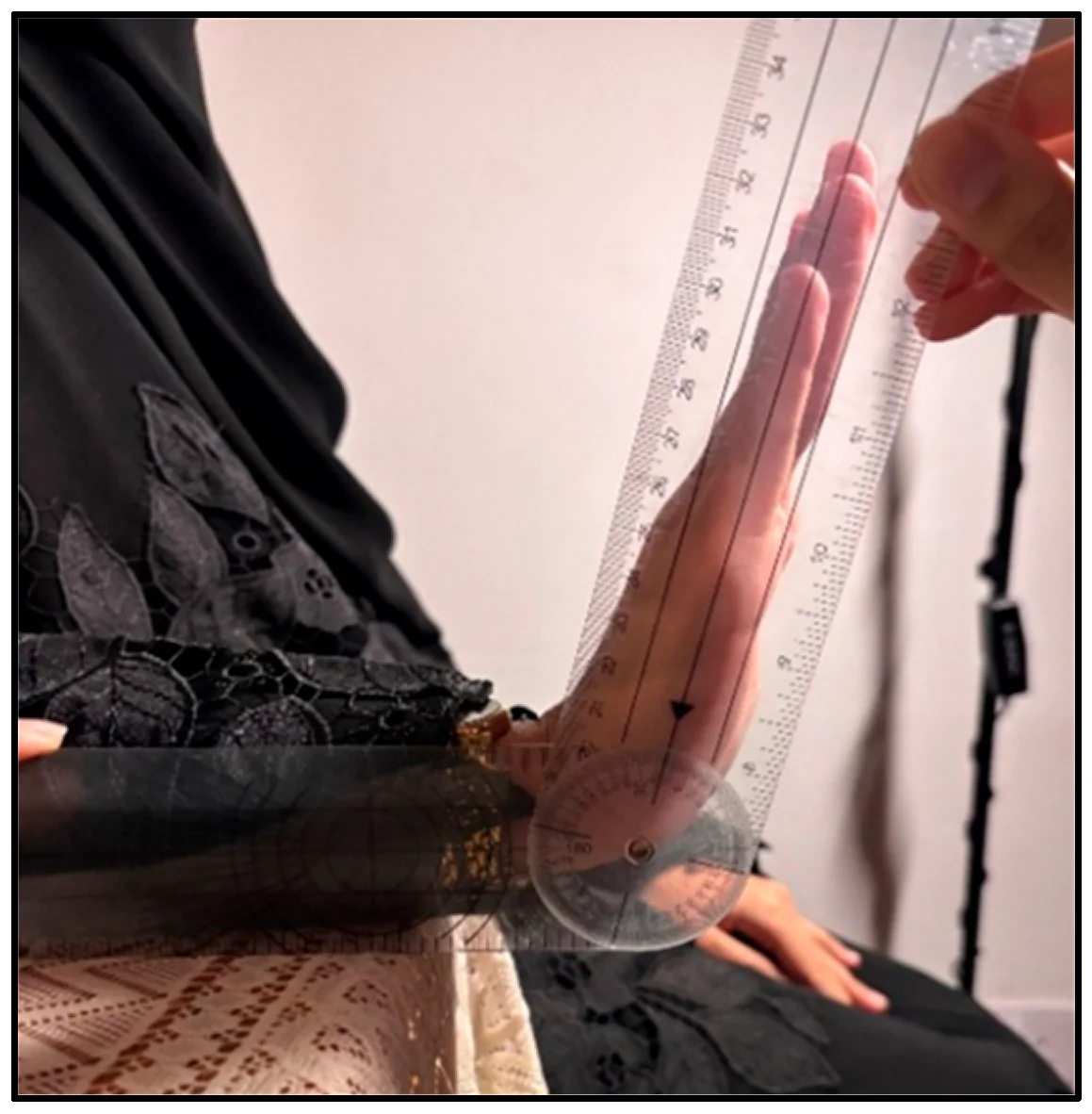
Manual goniometer used for assessing wrist extension.

**Figure 8:**
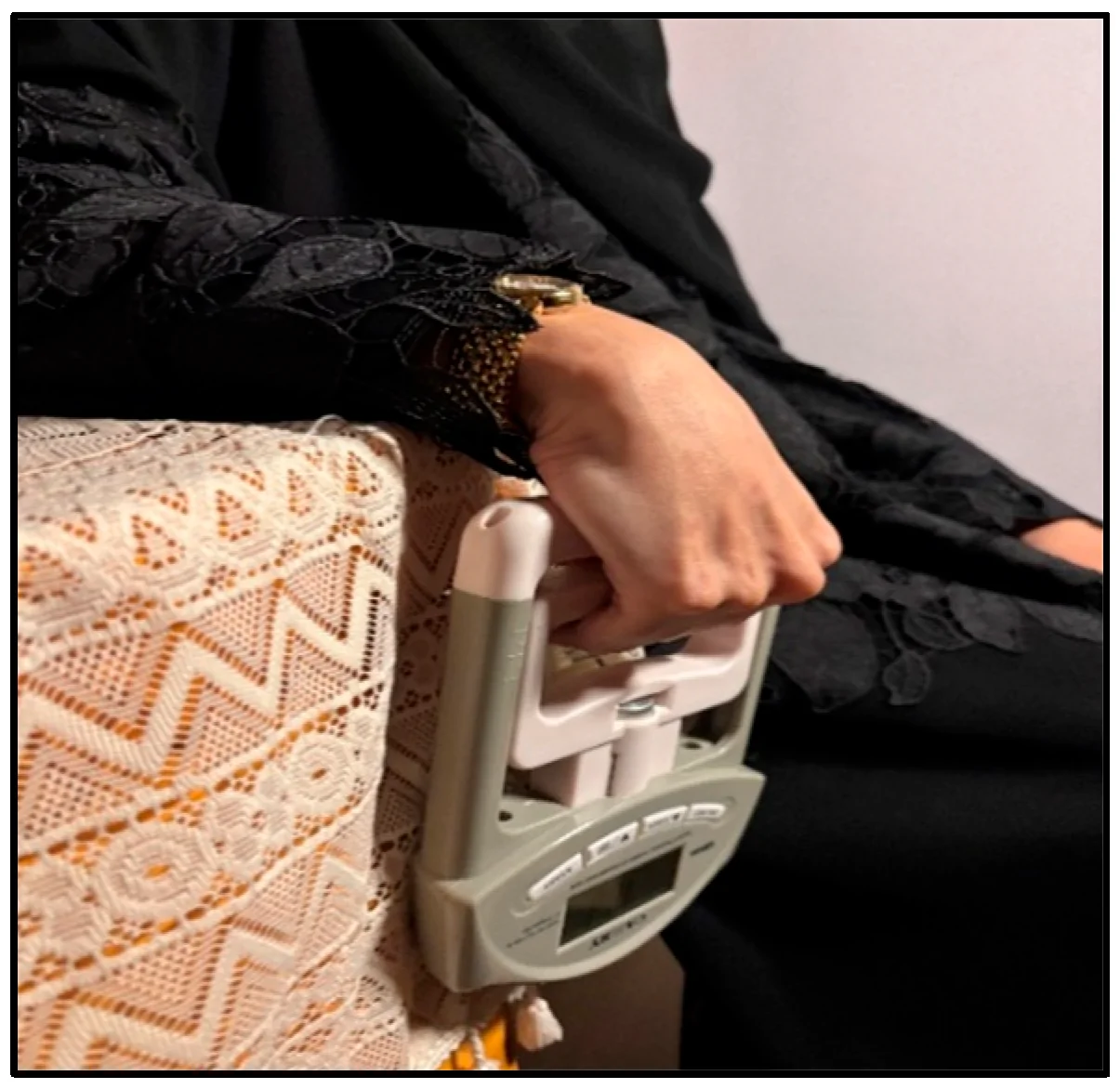
Measurement of wrist grip during flexion using a dynamometer.

**Figure 9:**
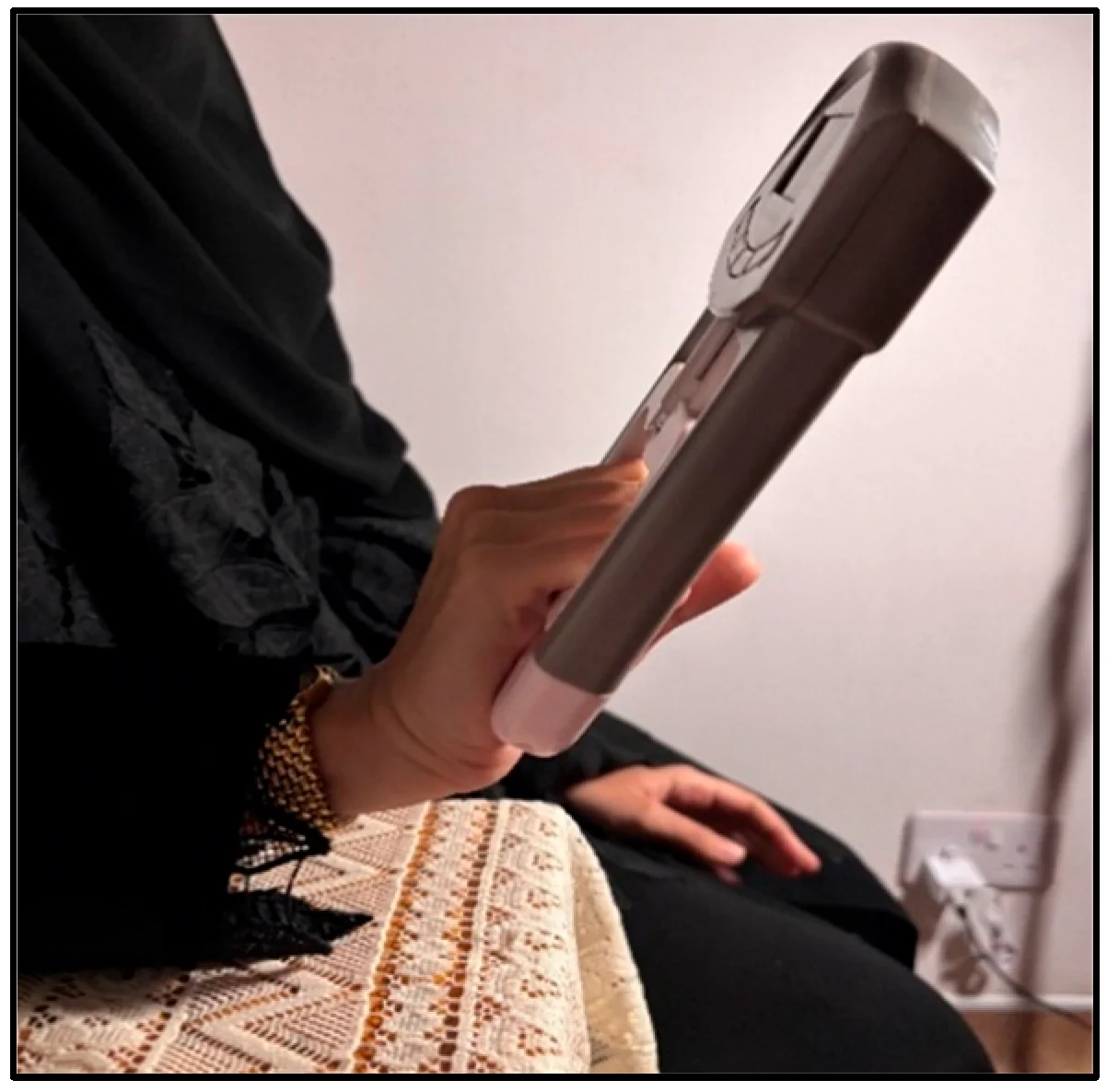
Measurement of wrist grip during wrist extension using a dynamometer.

For wrist flexion and extension measurements, the participants rested their forearms on a table while their wrists were positioned in a neutral starting position. The goniometer axis was placed over the triquetrum, the stationary arm was aligned with the ulna, and the moving arm was aligned with the fifth metacarpal bone. The participants actively performed maximum wrist flexion (Figure 6) and maximum wrist extension (Figure 7), and the corresponding angles were recorded. Grip strength measurements were then obtained during maximal wrist flexion (Figure 8), maximal wrist extension (Figure 9), and the neutral wrist position for comparison [18].

**Figure 10:**
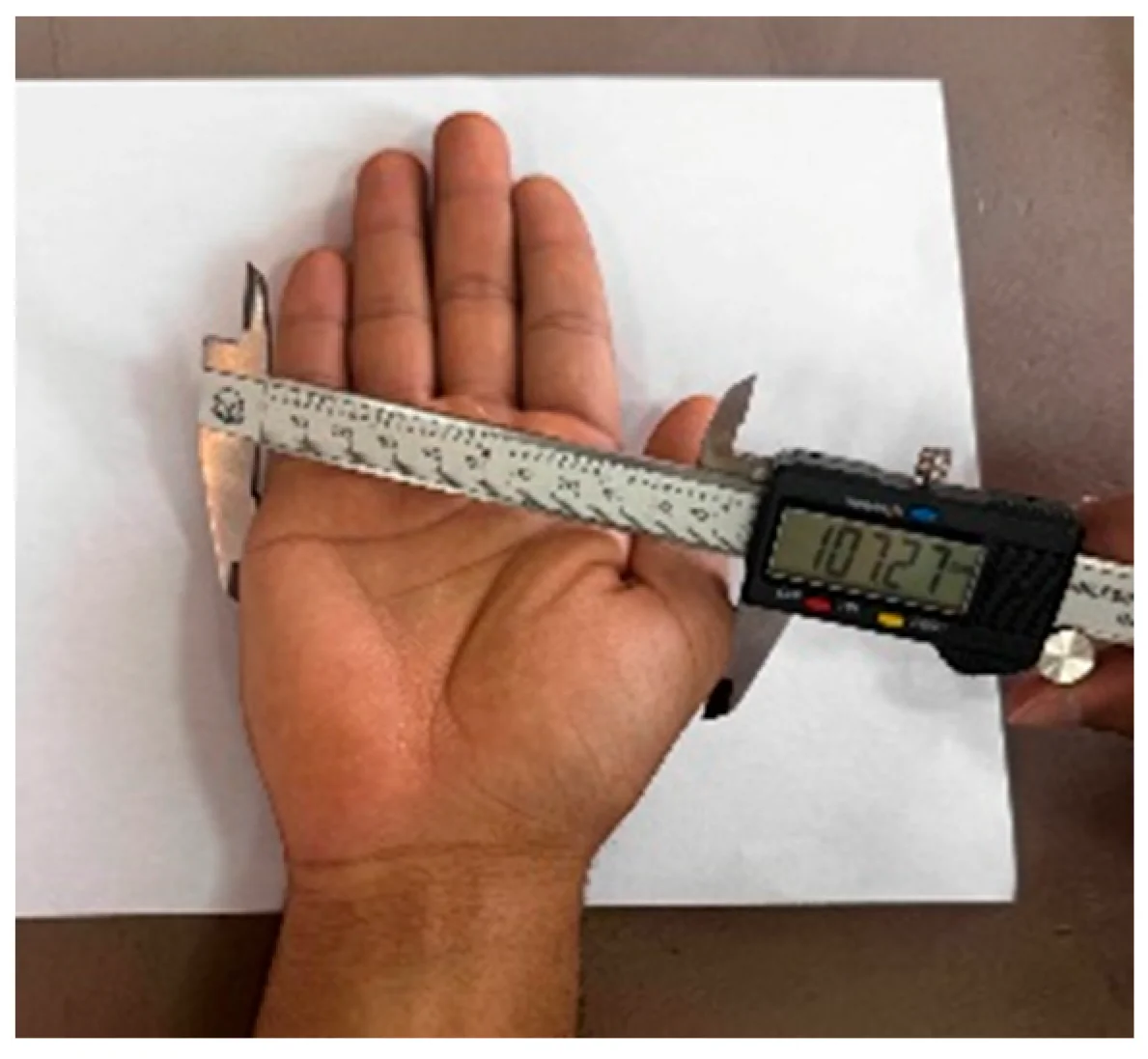
Digital caliper to measure hand anthropometric measurements.

**Figure 11:**
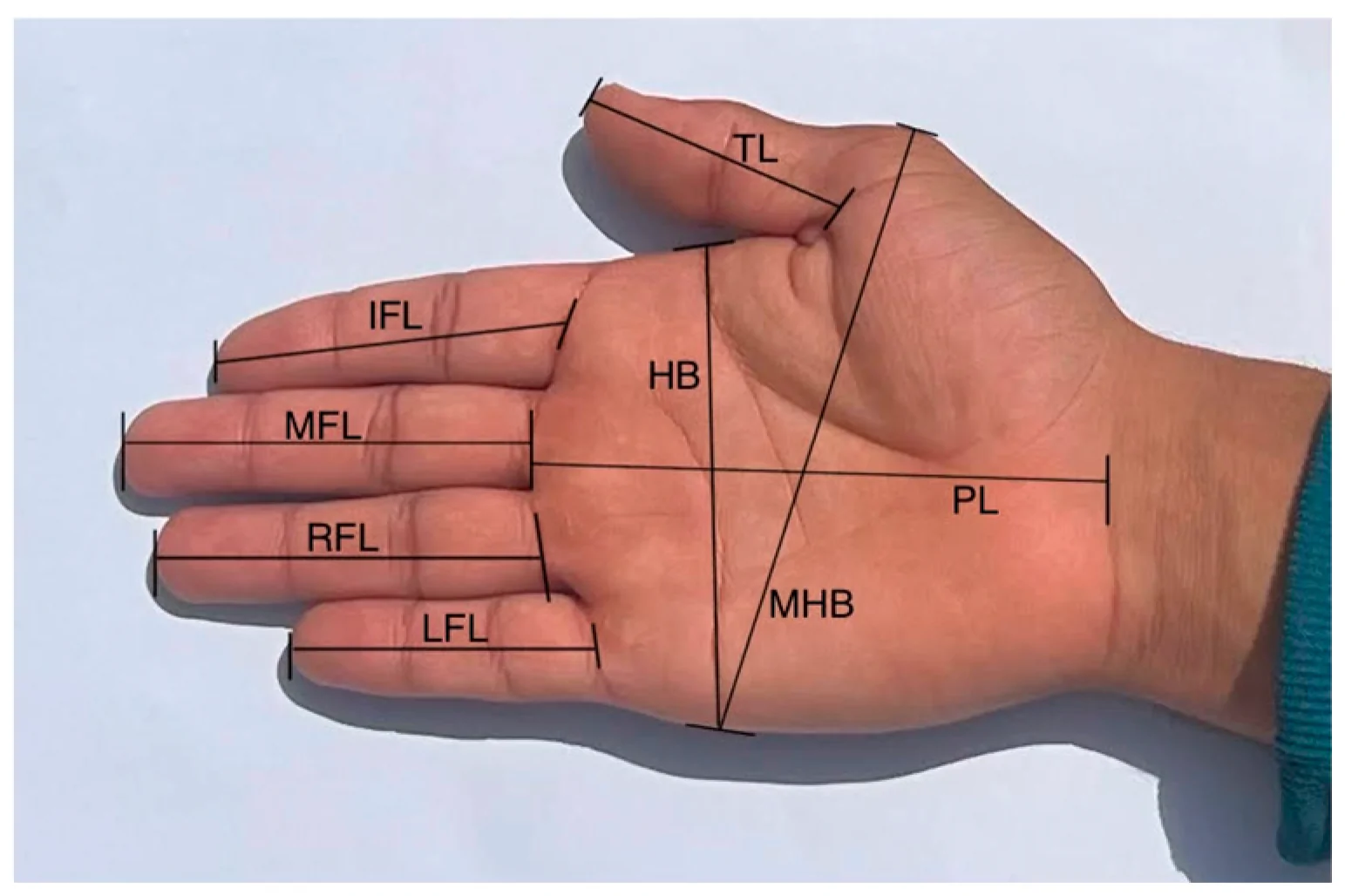
Hand anthropometric measurements. Note: thumb length (TL), index finger length (IFL), middle finger length (MFL), ring finger length (RFL), little finger length (LFL), hand breadth (HB), palm length (PL), maximum hand breadth (MHB).

Hand anthropometric measurements were obtained by having the participants place their dominant hand flat on a table with their fingers fully extended. A digital sliding caliper was used to measure the hand breadth (HB), maximum hand breadth (MHB), palm length (PL), thumb length (TFL), index finger length (IFL), middle finger length (MFL), ring finger length (RFL), and little finger length (LFL) (Figure 10). Standard anthropometric landmarks and measurement techniques were adopted, as described by Asadujjaman et al. (2019) [19] (Figure 11).

Data were collected after the participants provided informed consent and were briefed on the study objectives. Data analysis was performed using SPSS 26.0 software. Initially, the inter-observer reliability was assessed to evaluate the consistency of the measurements between the two observers. The Intraclass Correlation Coefficient (ICC) was used for this purpose.

This analysis used descriptive statistics for wrist ROM and grip strength, hand anthropometric measurements, and grip strength. Additionally, A mixed-design repeated-measures analysis of variance (ANOVA) was conducted to examine the effects of wrist position and sex on grip strength. Wrist position was treated as the within-subject factor and sex as the between-subject factor, and the interaction between wrist position and sex was also assessed. Where the assumption of sphericity was violated, the Greenhouse–Geisser correction was applied. Bonferroni-adjusted pairwise comparisons were performed to identify differences between wrist positions.

Correlation analyses were conducted to examine the relationships between wrist deviation measurements and grip strength at corresponding wrist positions, as well as relationships between grip strength measurements obtained at different wrist positions. Pearson’s and Spearman’s correlation coefficients were used, as appropriate, to assess the strength and direction of these associations. Spearman’s rank correlation analysis was also used to examine the relationships between wrist flexion and extension angles and grip strength at the corresponding wrist positions.

Spearman’s rank correlation analysis was performed to evaluate the relationships between grip strength and hand anthropometric measurements separately in males and females. Statistical significance was set at *p* < 0.05, and all statistical tests were two-tailed.

## 3. Results

### 3.1. Inter-Rater Reliability

Inter-rater reliability was assessed using the intraclass correlation coefficient (ICC) with a two-way random-effects model, absolute agreement, and single measurements [ICC (2,1)]. The corresponding 95% confidence intervals are presented in Table 1.

### 3.2. Demographic Characteristics

The study included an equal number of female and male participants. Each group comprised 81 individuals, representing 50% of the total study population (N = 162). All participants were aged 18–25 years.

### 3.3. Descriptive Statistics of Wrist Range of Motion and Grip Strength

**Figure 12:**
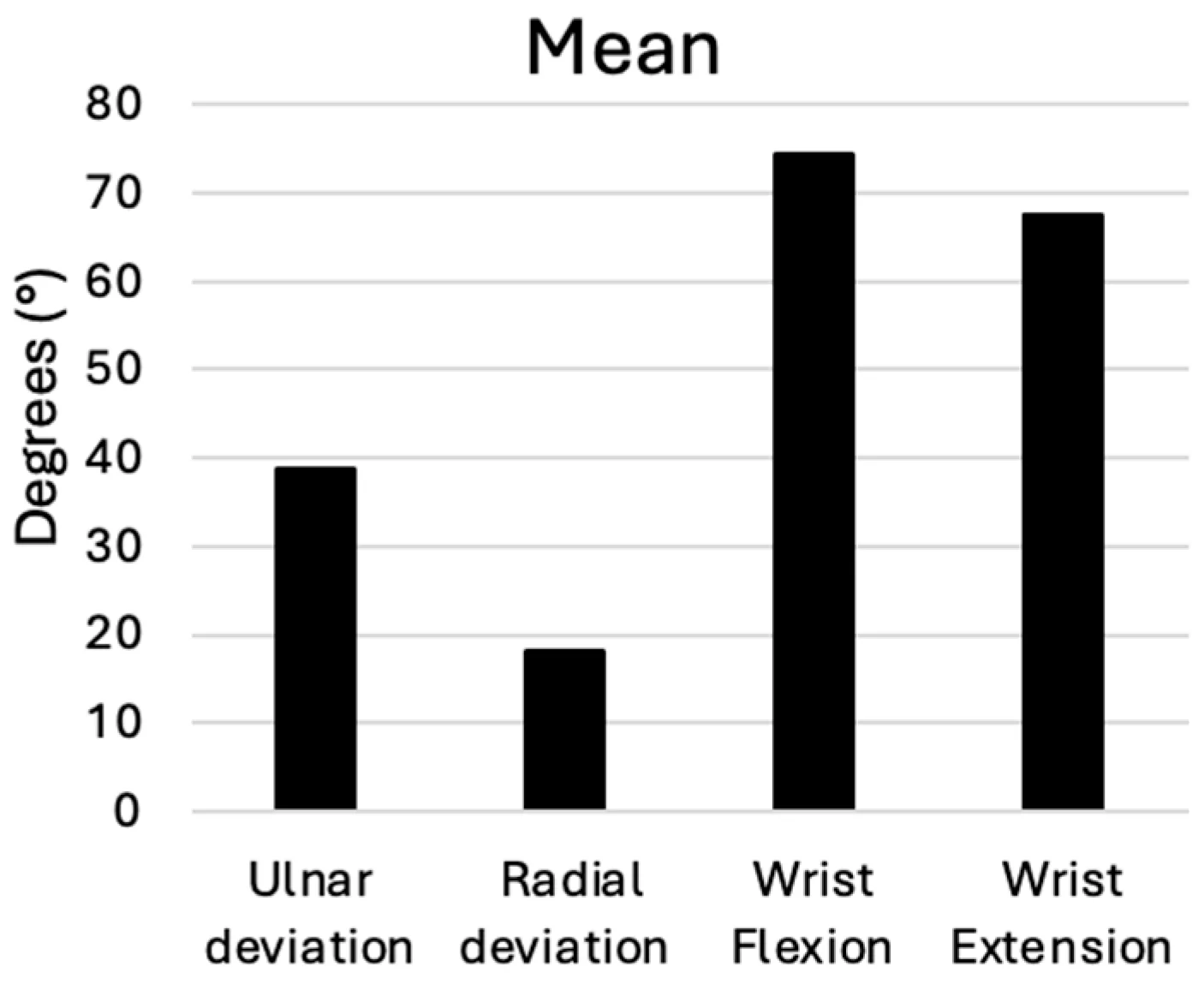
Mean wrist ROM.

The wrist ROM parameters were measured for all 162 participants, and mean values were calculated for the four measured motions: ulnar deviation, radial deviation, wrist flexion, and wrist extension. The results showed that the participants had the highest mean value at flexion of approximately 75°, followed by wrist extension at approximately 68°. In contrast, ulnar deviation showed a moderate mean value of approximately 39°, while radial deviation had the lowest mean value of approximately 19° (Figure 12).

**Figure 13:**
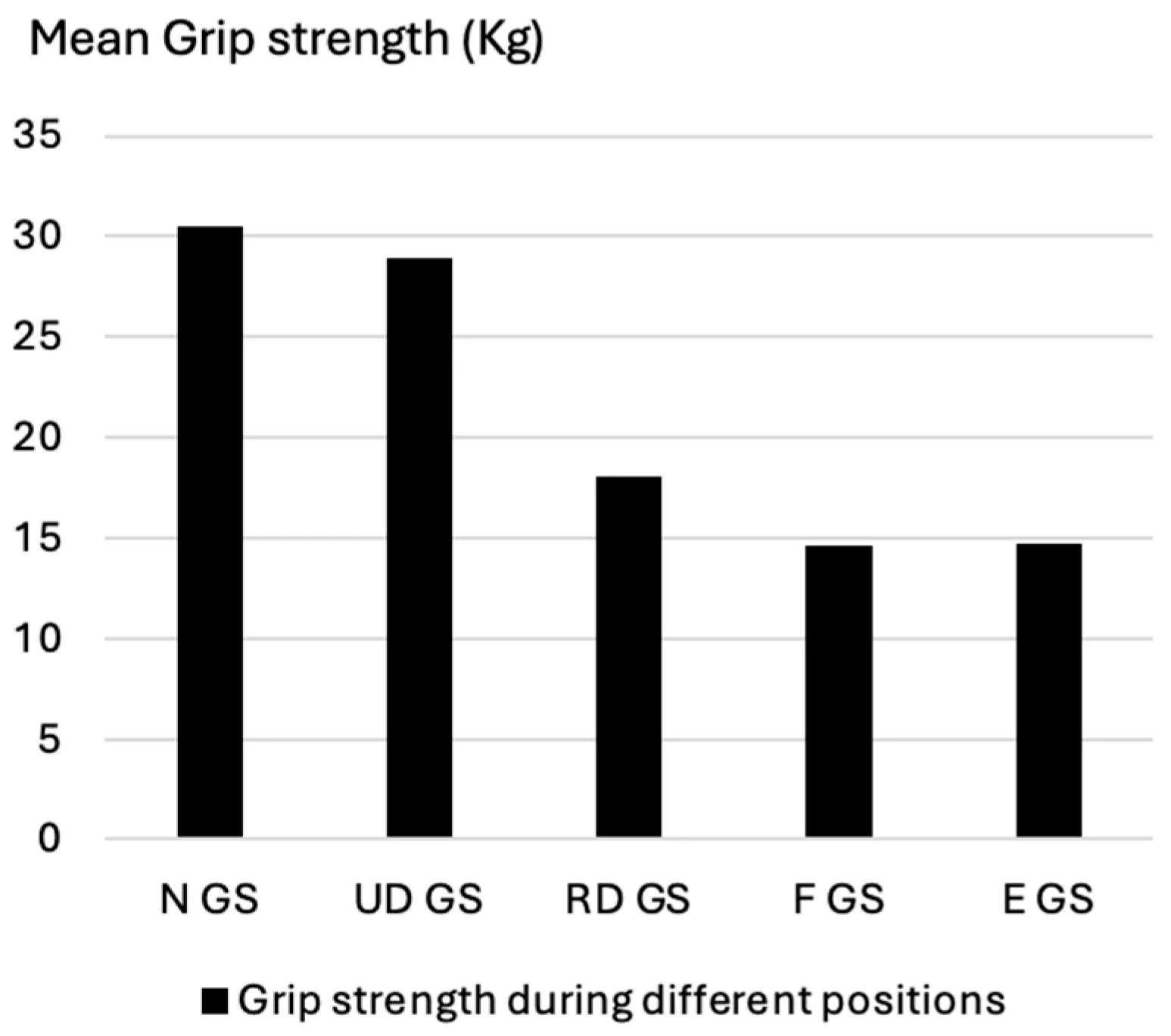
Mean grip strength during wrist ROM.

Grip strength was measured during the neutral position (N GS), ulnar deviation (UD GS), radial deviation (RD GS), flexion (F GS), and extension (E GS) positions. The mean values of grip strength in different positions were recorded (Figure 13). The participants recorded the highest grip strength during the neutral position (approximately 30.53 kg), followed closely by ulnar deviation (approximately 28.94 kg). Grip strength was lower in radial deviation (about 18.02 kg) and reached the lowest values during wrist flexion (about 14.67 kg) and wrist extension (about 14.75 kg).

### 3.4. Descriptive Statistics of Hand Anthropometric Measurements and Grip Strength

**Figure 14:**
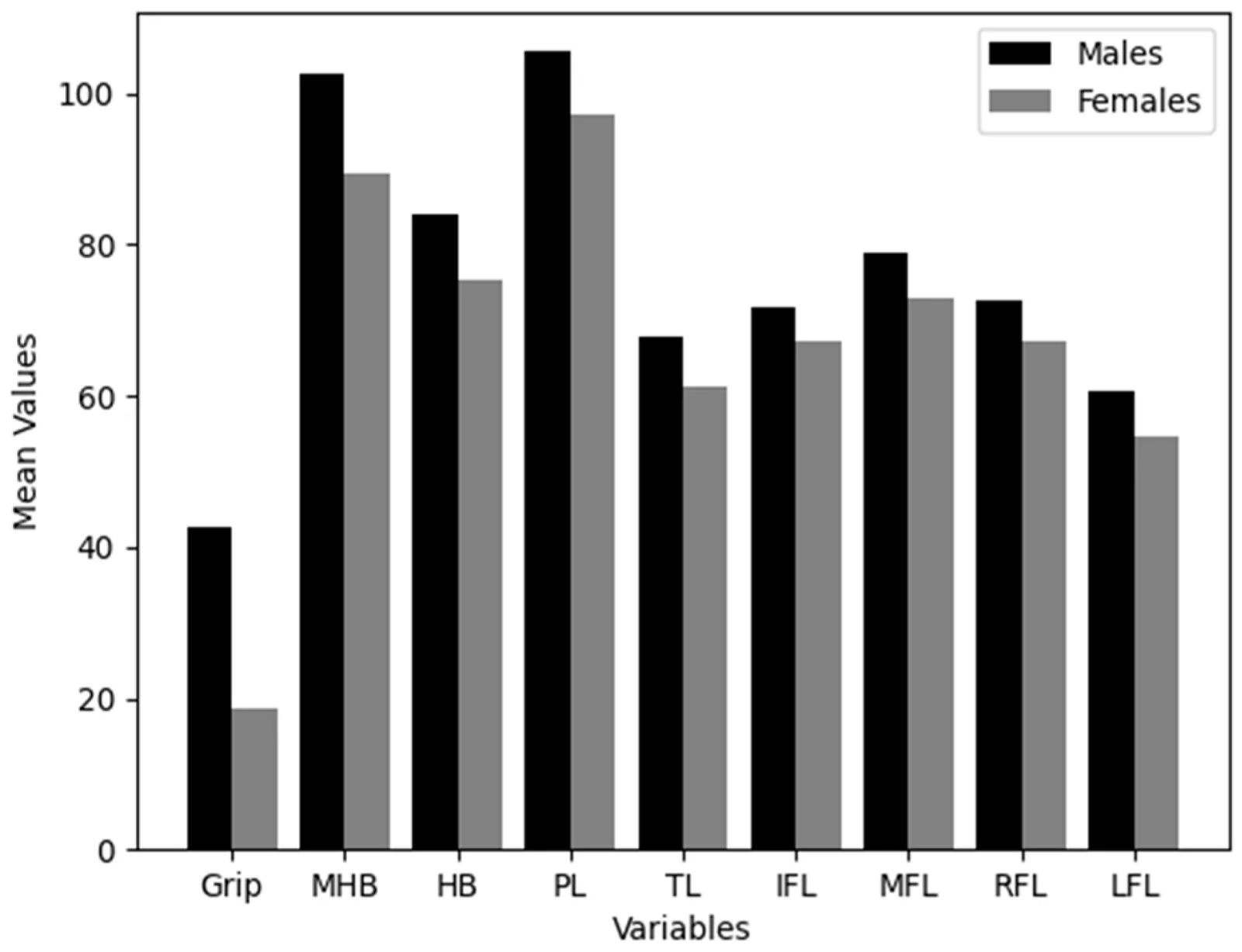
Mean values of hand anthropometric measurements and (GS) in males and females.

Descriptive analyses of hand anthropometric measurements and grip strength were performed separately for males and females (Figure 14). The mean grip strength was 42.56 (SD = 8.98) in males and 18.54 (SD = 4.20) in females, indicating a substantially greater grip strength in males. The mean maximum hand breadth (MHB) was 102.75 (SD = 5.95) in males and 89.34 (SD = 4.78) in females. The mean hand breadth (HB) was 83.90 (SD = 4.60) in males and 75.23 (SD = 4.44) in females. The mean palm length (PL) was 105.55 (SD = 5.47) in males and 97.11 (SD = 4.70) in females. Finger length measurements also showed higher mean values in males. The mean thumb length (TL) was 67.93 (SD = 4.71) in males and 61.21 (SD = 4.48) in females. The mean index finger length (IFL) was 71.80 (SD = 4.20) for males and 67.31 (SD = 4.20) for females. The mean middle finger length (MFL) was 78.88 (SD = 4.70) in males and 72.87 (SD = 5.12) in females. The mean ring finger length (RFL) was 72.67 (SD = 4.47) in males and 67.35 (SD = 5.46) in females. The mean little finger length (LFL) was 60.71 (SD = 4.05) in males and 54.51 (SD = 3.87) in females.

### 3.5. Wrist Deviations and Grip Strength

Correlation analyses were conducted to examine the relationships between wrist deviation measurements and grip strength at corresponding wrist positions, as well as relationships between grip strength measurements obtained at different wrist positions. Pearson’s and Spearman’s correlation coefficients were used to assess the strength and direction of these associations, respectively.

As shown in Table 2, positive associations were observed between wrist deviation and grip strength measurements. The strongest association was observed between ulnar deviation grip strength (UD GS) and neutral position grip strength (N GS), with a Spearman correlation coefficient of 0.81 and a Pearson correlation coefficient of 0.79. Radial deviation grip strength (RD GS) was also strongly correlated with neutral-position grip strength (Spearman ρ = 0.76; Pearson r = 0.71). A strong positive association was also observed between radial deviation and grip strength (Spearman ρ = 0.78; Pearson r = 0.74). In comparison, the association between UD GS and RD GS was weaker, although it was still positive (Spearman ρ = 0.50; Pearson r = 0.40). Overall, grip strength measurements obtained at different wrist positions demonstrated positive associations.

### 3.6. Wrist Flexion and Extension Degrees and Grip Strength

Spearman’s rank correlation coefficient was used to examine the relationships between wrist flexion and extension angles and grip strength at the corresponding wrist positions, as well as the relationships between grip strength measurements obtained at different wrist positions (Table 3).

A weak negative association was observed between the wrist flexion angle and grip strength during flexion (ρ = −0.24, *p* < 0.05), whereas a moderate negative association was observed between the wrist extension angle and grip strength during extension (ρ = −0.41, *p* < 0.05). Strong positive associations were observed among grip strength measurements across wrist positions. Grip strength during flexion was strongly correlated with the grip strength during extension (ρ = 0.82, *p* < 0.05) and neutral-position grip strength (ρ = 0.84, *p* < 0.05). Similarly, grip strength during extension was strongly correlated with neutral-position grip strength (ρ = 0.83, *p* < 0.05).

### 3.7. Effect of Wrist Position and Sex on Grip Strength

A mixed-design repeated-measures analysis of variance (ANOVA) was conducted to examine the effects of wrist position and sex on grip strength. Wrist position was treated as a within-subject factor with five levels (neutral, flexion, extension, ulnar deviation, and radial deviation), while sex was treated as a between-subject factor. Mauchly’s test indicated a significant violation of the sphericity assumption (W = 0.581, χ^2^(9) = 85.926, *p* < 0.001); therefore, the Greenhouse–Geisser correction was applied to the within-subject effects.

There was a statistically significant main effect of wrist position on grip strength, F(3.05, 487.95) = 532.62, *p* < 0.001, partial η^2^ = 0.769. A statistically significant main effect of sex was also observed, F(1, 160) = 436.04, *p* < 0.001, partial η^2^ = 0.732. In addition, a statistically significant interaction between wrist position and sex was observed, F(3.05, 487.95) = 138.22, *p* < 0.001, partial η^2^ = 0.463 (Table 4).

Bonferroni-adjusted pairwise comparisons demonstrated that grip strength in the neutral position was significantly higher than that in flexion, extension, ulnar deviation, and radial deviation (all *p* < 0.001). No significant differences were observed between flexion and extension (*p* = 1.000) or between ulnar and radial deviation (*p* = 1.000). Significant differences were observed between flexion and ulnar deviation, flexion and radial deviation, extension and ulnar deviation, and extension and radial deviation (all *p* < 0.001) (Table 5).

Across all five wrist positions, males demonstrated higher grip strength values than females. The mean grip strength in the neutral position was 42.56 ± 8.98 kg among males and 18.54 ± 4.20 kg among females. The corresponding mean values for flexion, extension, ulnar deviation, and radial deviation were 18.94 ± 4.57, 19.17 ± 4.84, 22.33 ± 5.02, and 22.49 ± 6.05 kg in males, compared to 10.41 ± 2.37, 10.34 ± 2.40, 12.76 ± 2.64, and 13.24 ± 4.18 kg in females.

### 3.8. Hand Anthropometric Measurements

Spearman’s rank correlation analysis was performed to evaluate the relationship between GS and hand anthropometric measurements in males and females (Table 6).

In males, statistically positive correlations were observed between grip strength and several hand anthropometric measurements. The strongest correlation was observed with maximum hand breadth (MHB) (ρ = 0.365, *p* = 0.001), followed by hand breadth (HB) (ρ = 0.326, *p* = 0.003) and palm length (PL) (ρ = 0.304, *p* = 0.006) measurements. Weak positive correlations were also observed with thumb length (TL) (ρ = 0.289, *p* = 0.009) and index finger length (IFL) (ρ = 0.229, *p* = 0.039). In contrast, the correlations between grip strength and middle finger length (MFL) (ρ = 0.215, *p* = 0.054), ring finger length (RFL) (ρ = 0.112, *p* = 0.320), and little finger length (LFL) (ρ = 0.126, *p* = 0.261) were not statistically significant.

In females, no statistically significant correlations were observed between grip strength and any of the assessed hand anthropometric measurements. The correlations were weak and non-significant for MHB (ρ = −0.005, *p* = 0.962), HB (ρ = −0.076, *p* = 0.501), PL (ρ = −0.112, *p* = 0.320), TL (ρ = −0.056, *p* = 0.620), IFL (ρ = −0.202, *p* = 0.071), MFL (ρ = −0.193, *p* = 0.085), RFL (ρ = −0.138, *p* = 0.219), and LFL (ρ = −0.158, *p* = 0.158).

## 4. Discussion

This study aimed to investigate the effect of different wrist positions on grip strength and the relationship between grip strength and hand anthropometric measurements in males and females. These findings provide insights into the influence of wrist position and sex on grip strength and the associations between wrist characteristics, grip strength, and hand anthropometric measurements.

Measuring wrist ROM is a fundamental component of physical examination and plays an important role in evaluating, diagnosing, treating, and monitoring patients with wrist disorders [20]. Furthermore, adequate wrist ROM is essential for optimal upper limb function and performance of activities of daily living [6]. Compared with a previous study conducted on 52 healthy individuals, mean values of 74.4° for flexion, 71.1° for extension, 19.7° for radial deviation, and 34.0° for ulnar deviation were reported [21], which are comparable to the findings of the present study. Previous research has also reported sex-related differences in wrist ROM, with females demonstrating a greater ROM for some wrist movements than males [22]. In the present study, female participants demonstrated greater mean flexion and extension ROM, whereas male participants demonstrated higher mean values for radial and ulnar deviation.

The present study demonstrated a significant effect of wrist position on grip strength, as well as significant effects of sex and the interaction between wrist position and sex. In the present study, wrist ulnar and radial deviations and grip strength were intrinsically linked by significant positive correlations. The significant wrist position × sex interaction indicates that the pattern of grip strength changes across wrist positions differed between males and females, which is consistent with normative population studies reporting that males generally exhibit greater grip strength than females across all adult age groups [23]. These findings are consistent with those of Lee and Sechachalam (2016) [8], who reported that grip strength is influenced by wrist position, and grip performance varies according to wrist posture. Furthermore, our observation that grip strength was greater during ulnar deviation than during radial deviation is supported by Popp et al. (2023) [24], who demonstrated that wrist posture significantly affects grip force production and that non-optimal wrist positions reduce force generation. In addition, male participants produced grip strength that was 2.5 to 3 times higher than female strength, which can be illustrated by physiological and muscle mass differences between the sexes [25].

This present study investigated the relationship between wrist joint position (flexion and extension) and grip strength in healthy individuals. The findings showed a negative correlation between the two conditions, indicating that as the wrist joint angle increased, the grip strength decreased. In flexion, the correlation was weak but statistically significant (ρ = −0.24, *p* < 0.05). This means that during flexion, the muscle length and mechanical advantage may slightly reduce the power capacity to produce maximal grip force, which agrees with a study conducted by Bhardwaj et al. (2011) [26], which indicated that grip strength is significantly reduced during flexion. For extension, the correlation was moderate and statistically significant (ρ = −0.41, *p* < 0.005), indicating a stronger inverse relationship than that observed for flexion. This suggests that an increase in the extension angle has a greater influence on reducing grip strength. This may be due to the decreased performance capacity of the flexor muscles when the wrist is extended.

Our findings showed very strong and statistically significant positive correlations among grip strength in the flexion, extension, and normal wrist positions. This demonstrates that grip strength is highly reliable across various wrist positions. These strong correlations suggest that grip strength is primarily affected by overall muscle strength, rather than by the specific position of the wrist. Hence, individuals with stronger muscle capacity can achieve higher grip strength regardless of wrist position. Similar observations were reported by Lee and Sechachalam (2016) [8], who found that grip strength varied with wrist position but remained highest in participants with greater overall strength, regardless of the tested position.

The present study also demonstrated a significant main effect of sex on grip strength, with males demonstrating higher grip strength values than females in all tested wrist positions. This finding is consistent with previous population-based studies that have shown substantial sex-related differences in grip strength [23,25]. Napper et al. [27] reported consistent sex differences in wrist strength. These differences may be related to variations in anthropometric characteristics between males and females. Chaparro et al. [28] reported sex-related differences in wrist range of motion, including greater extension ROM in females. This is broadly consistent with the present finding that females demonstrated greater flexion and extension ROM, which is similar to our study, indicating that females scored higher degrees of flexion and extension measurements [28]. This indicates that females have a greater ROM and more flexibility, whereas males tend to demonstrate greater muscular strength because of their biological differences. Importantly, the significant wrist position with respect to sex indicated that the influence of wrist position on grip strength differed between males and females. Therefore, the effect of wrist posture cannot be interpreted independently of sex. Although males demonstrated higher grip strength across all tested positions, the pattern of change across wrist positions was not identical between the two groups. This finding supports the use of a repeated-measures analytical approach that accounts for both within-participant changes across wrist positions and between-sex differences.

The results revealed significant differences between the sexes in the correlation between GS and hand anthropometric measurements. GS was associated with several hand measurements in males. MHB and HB showed moderate correlations with GS, indicating that wider hands tend to have stronger grip strength. PL, TL, and IFL also exhibited weaker relationships with each other. In contrast, GS was not overtly related to MFL, RFL, and LFL. This implies that overall hand size and certain finger lengths are important for determining GS. These findings are consistent with those of previous studies that reported a significant relationship between hand dimensions and grip strength [29].

The results for females were different. No evident relationship was observed between GS and any hand measurements. All correlations were weak and insignificant. This indicates that GS is not influenced by hand size in females. Similar findings have been reported in other studies, where grip strength was influenced by multiple factors beyond anthropometric measurements [30]. Compared with both groups, hand measurements had a greater influence on grip strength in males than in females. This may be due to other factors; however, this is beyond the scope of our study. The results are important for grip strength in males and can be estimated using hand measurements.

Grip strength has many clinical implications. Grip strength differentiates older individuals based on their mobility, even though it is not directly necessary for performing functional tasks such as walking [2]. Previous studies have revealed limited grip strength in individuals with diabetes [31,32,33] or prediabetes [34]. Handgrip strength has been reported to be associated with multiple chronic diseases after controlling for comorbidities, including not only the muscular system but also disorders and diseases of other systems. These include anemia, anxiety, chronic kidney disease of stage 3 or beyond, chronic obstructive pulmonary disease, hyperthyroidism, stroke, and kyphosis [35]. Another study showed that isometric handgrip training for 5–6 weeks reduced resting arterial blood pressure [36].

Negative ulnar variance or a short ulna has clinical implications in Kienbock’s disease, avascular necrosis of the scaphoid, and scapho-lunate dissociations [37,38,39]. In contrast, positive ulnar variance predisposes the wrist to triangular fibrocartilaginous cartilage complex (TFCC) injury and early degenerative arthritis of the wrist [40,41].

The range of radioulnar deviation denotes the flexibility of the radioulnar carpal joint [42]. A previous study found that the radial deviation of the left hand in manual workers was statistically significantly different from that in non-manual workers, implying greater flexibility for manual workers [42].

In clinical practice, it is important to observe wrist movements properly. Ulnar drift of the fingers and radial deviation of the wrist can occur in rheumatoid arthritis, further impairing hand function and grip strength [43]. Patients with distal radial fractures have a limited range of ROM. The ROM in different planes of movement includes wrist flexion and extension, wrist radial deviation, and ulnar deviation [7]. Hence, occupational therapists can design appropriate rehabilitation strategies accordingly.

The findings of this study can also be useful in daily life settings. For example, hand measurements could help estimate grip strength in males in clinical or rehabilitation settings. However, other factors should be considered when evaluating grip strength in females.

The present study had some limitations that should be considered when interpreting the results. One of the limitations is that the sample participants were all SQU medical students in the age group of 18–25 years. We admit that this can affect and limit the diversity of the sample and its generalizability to the Omani population. Additionally, as a pilot, cross-sectional study, the present findings should be interpreted with caution. There were some difficulties in simultaneously measuring grip strength at different wrist positions at the same time. Time of day was not controlled, as data were collected from each participant at different times, as mentioned in Section 2. These factors may have influenced our results. Therefore, when applying these results and findings in clinical practice or academic contexts, these limitations should be considered.

## 5. Conclusions

This study explored the effects of different wrist positions on grip strength and examined the association between grip strength and hand anthropometric measurements in male and female participants separately. The findings showed that wrist position had a clear influence on grip strength, with the highest values produced in the neutral position and the lowest values produced during wrist flexion and extension. Grip strength during ulnar deviation was also higher than during radial deviation, and this is similar to past studies. The negative correlations found between flexion and extension angles and their corresponding grip strengths further support the idea that the grip force decreases as the wrist moves away from its neutral position, with extension showing a stronger inverse relationship than flexion.

The findings of the present study can be useful in several practical fields, including rehabilitation, occupational therapy, sports performance, and ergonomic design of workspaces. Because grip strength drops when the wrist moves away from the neutral position, this information can be helpful for therapists and trainers when planning exercises or daily activities, so the chance of injury is lowered and hand function is improved. Grip strength and wrist position during ulnar and radial deviation have enormous biomechanical and clinical relevance, as they are needed for optimal hand performance. Grip strength may not only be associated with musculoskeletal disorders but also with other disorders in the body. Hence, a meticulous physical examination is warranted for such patients. Radial and ulnar deviations following fractures and in association with rheumatoid disease could help plan better rehabilitation strategies.

For males, hand measurements can provide a useful estimate of grip strength in clinical practice; however, for females, other factors need to be considered because hand size alone does not provide a clear answer to this question. The results of the present study may benefit individuals undergoing various rehabilitation programs associated with ligamentous injuries of the wrist, triangular fibrocartilage complex injuries, tendon repair, and arthroplasty of the wrist. Occupational therapists may restore controlled radial and ulnar deviation to enhance dynamic stability and hand function.

## Figures and Tables

**Table 1:** Inter-rater reliability of wrist measurements, GS, and hand anthropometric measurements.

Variable	Male ICC (95% CI)	Female ICC (95% CI)
UD (°)	0.434 (0.225–0.601)	0.784 (0.684–0.855)
UD GS	0.434 (0.221–0.603)	0.718 (0.594–0.809)
RD (°)	0.360 (0.070–0.575)	0.817 (0.729–0.878)
RD GS	0.577 (0.411–0.705)	0.891 (0.835–0.928)
F (°)	0.461 (0.221–0.637)	0.825 (0.740–0.883)
F GS	0.631 (0.480–0.746)	0.843 (0.766–0.896)
E (°)	0.323 (0.066–0.529)	0.760 (0.650–0.839)
E GS	0.716 (0.589–0.809)	0.782 (0.680–0.854)
MHB	0.816 (0.727–0.877)	0.811 (0.721–0.874)
HB	0.889 (0.675–0.949)	0.708 (0.580–0.802)
PL	0.686 (0.532–0.793)	0.805 (0.713–0.870)
TL	0.739 (0.404–0.869)	0.691 (0.555–0.790)
IFL	0.893 (0.823–0.934)	0.901 (0.850–0.935)
MFL	0.952 (0.927–0.969)	0.926 (0.888–0.952)
RFL	0.930 (0.888–0.956)	0.924 (0.884–0.950)
LFL	0.896 (0.840–0.933)	0.890 (0.834–0.917)
N GS	0.802 (0.709–0.868)	0.946 (0.917–0.965)

Note: ulnar deviation (UD), grip strength during ulnar deviation (UD GS), radial deviation (RD), grip strength during radial deviation (RD GS), flexion (F), grip strength during flexion (F GS), Extension (E), grip strength during extension (E GS), maximum hand breadth (MHB), hand breadth (HB), palm length (PL), thumb length (TL), index finger length (IFL), middle finger length (MFL), ring finger length (RFL), little finger length (LFL).

**Table 2:** Correlation between wrist deviations and grip strength.

UD vs. N GS	Value	Approx. T
Spearman Correlation	0.64	10.64
Pearson’s R	0.55	8.36
RD vs. N GS		
Spearman Correlation	0.78	15.85
Pearson’s R	0.74	14.05
UD GS vs. RD GS		
Spearman Correlation	0.50	7.25
Pearson’s R	0.40	5.47
UD GS vs. N GS		
Spearman Correlation	0.81	17.68
Pearson’s R	0.79	16.12
RD GS vs. N GS		
Spearman Correlation	0.76	14.60
Pearson’s R	0.71	12.77

Note: ulnar deviation (UD), radial deviation (RD), grip strength during ulnar deviation (UD GS), grip strength during radial deviation (RD GS), and Grip strength during the neutral position (N GS).

**Table 3:** Correlation between wrist flexion and extension and grip strength.

F vs. N GS	Value	Approx. T
Spearman Correlation	−0.24	−3.20
Pearson’s R	−0.30	−4.05
E vs. N GS		
Spearman Correlation	−0.41	−5.777
Pearson’s R	−0.36	−4.84
F GS vs. F GS		
Spearman Correlation	0.82	18.28
Pearson’s R	0.78	15.77
F GS vs. N GS		
Spearman Correlation	0.84	19.42
Pearson’s R	0.82	18.27
E GS vs. N GS		
Spearman Correlation	0.83	19.15
Pearson’s R	0.80	16.97

Note: Flexion (F), grip strength during flexion (F GS), Extension (E), grip strength during extension (E GS) and Grip strength during the neutral position (N GS).

**Table 4:** Mixed-design repeated-measures ANOVA examining the effects of wrist position, sex, and their interaction on grip strength.

Effect	F	df	*p*-Value	Partial η^2^
Wrist position	532.62	3.05, 487.95	<0.001	0.769
Sex	436.04	1, 160	<0.001	0.732
Wrist position and sex interaction	138.22	3.05, 487.95	<0.001	0.463

**Table 5:** Bonferroni-adjusted pairwise comparisons of grip strength across wrist positions, stratified by sex.

Sex	Wrist-Position Comparison	Mean Difference (kg)	Adjusted *p*-Value
Female	N GS vs. F GS	8.13	<0.001
	N GS vs. E GS	8.20	<0.001
	N GS vs. UD GS	5.78	<0.001
	N GS vs. RD GS	5.29	<0.001
	F GS vs. E GS	0.07	1.000
	F GS vs. UD GS	−2.35	<0.001
	F GS vs. RD GS	−2.84	<0.001
	E GS vs. UD GS	−2.69	<0.001
	E GS vs. RD GS	−3.01	<0.001
	UD GS vs. RD GS	−0.16	1.000
Male	N GS vs. F GS	20.23	<0.001
	N GS vs. E GS	20.07	<0.001
	N GS vs. UD GS	23.62	<0.001
	N GS vs. RD GS	23.39	<0.001
	F GS vs. E GS	−0.23	1.000
	F GS vs. UD GS	−3.39	<0.001
	F GS vs. RD GS	−3.55	<0.001
	E GS vs. UD GS	−3.16	<0.001
	E GS vs. RD GS	−3.32	<0.001
	UD GS vs. RD GS	−0.16	1.000

Note: Grip strength during the neutral position (N GS), grip strength during ulnar deviation (UD GS), grip strength during radial deviation (RD GS), grip strength during flexion (F GS), and grip strength during extension (E GS).

**Table 6:** Comparison of grip strength correlation with hand anthropometric measurements between males and females.

Variable	Males (ρ)	*p*-Value	Females (ρ)	*p*-Value
MHB	0.365	0.001	−0.005	0.962
HB	0.326	0.003	−0.076	0.501
PL	0.304	0.006	−0.112	0.320
TL	0.289	0.009	−0.056	0.620
IFL	0.229	0.039	−0.202	0.071
MFL	0.215	0.053	−0.193	0.085
RFL	0.112	0.320	−0.138	0.219
LFL	0.126	0.261	−0.158	0.158

Note: maximum hand breadth (MHB), hand breadth (HB), palm length (PL), thumb length (TL), index finger length (IFL), middle finger length (MFL), ring finger length (RFL), and little finger length (LFL).

## Data Availability

The data presented in this study are available on request from the corresponding author. Due to ethical and legal restrictions, the data supporting this study are not publicly available.

## Abbreviations

The following abbreviations are used in this manuscript:

SQU	Sultan Qaboos University
UD	Ulnar deviation
RD	Radial Deviation
F	Flexion
E	Extension
GS	Grip strength
HB	Hand Breadth
MHB	Maximum Hand Breadth
PL	Palm Length
TFL	Thumb Length
IFL	Index Finger Length
MFL	Middle Finger Length
RFL	Ring Finger Length
LFL	Little Finger Length

## References

[1] Vaishya R., Misra A., Vaish A., Ursino N., D’Ambrosi R. (2024). Hand grip strength as a proposed new vital sign of health: A narrative review of evidences. J. Health Popul. Nutr..

[2] Bohannon R.W. (2019). Grip strength: An indispensable biomarker for older adults. Clin. Interv. Aging.

[3] Norman K., Stobäus N., Gonzalez M.C., Schulzke J.D., Pirlich M. (2011). Hand grip strength: Outcome predictor and marker of nutritional status. Clin. Nutr..

[4] Garcia-Carrillo E., Salas-Gómez D., Castillo-Paredes A., Becerra-Patiño B.A., Farías-Valenzuela C., Cortés-Roco G., Alarcón-Rivera M., Fuentes-Barría H., Yáñez-Sepúlveda R. (2026). Mapping handgrip strength research in sports performance: A bibliometric review of applications, trends, and future directions. Sports.

[5] Roby-Brami A., Jarrassé N., Parry R. (2021). Impairment and compensation in dexterous upper-limb function after stroke: From the direct consequences of pyramidal tract lesions to behavioral involvement of both upper limbs in daily activities. Front. Hum. Neurosci..

[6] Rainbow M.J., Wolff A.L., Crisco J.J., Wolfe S.W. (2016). Functional kinematics of the wrist. J. Hand Surg. Eur. Vol..

[7] Yang Z., Lim P.P.H., Teo S.H., Chen H., Qiu H., Pua Y.H. (2018). Association of wrist and forearm range of motion measures with self-reported functional scores amongst patients with distal radius fractures: A longitudinal study. BMC Musculoskelet. Disord..

[8] Lee J.-A., Sechachalam S. (2016). The effect of wrist position on grip endurance and grip strength. J. Hand Surg. Am..

[9] Hong S.-J., Lee M.-Y., Lee B.-H. (2024). Effects of wrist stability training combined with grip strength exercise on pain and function in patients with nonspecific chronic wrist pain. Medicina.

[10] Quattrocchi A., Garufi G., Gugliandolo G., De Marchis C., Collufio D., Cardali S.M., Donato N. (2024). Handgrip Strength in Health Applications: A Review of the Measurement Methodologies and Influencing Factors. Sensors.

[11] Cohen J. (1988). Statistical Power Analysis for the Behavioral Sciences.

[12] Faul F., Erdfelder E., Lang A.-G., Buchner A. (2007). G*Power 3: A flexible statistical power analysis program for the social, behavioral, and biomedical sciences. Behav. Res. Methods.

[13] Zea Forero C.R., Medina-Labrador M., Monroy Silva M., Jimenez Gordillo J.-F. (2025). An Analysis of Hand Size and Grip Strength in a Working Population in Bogotá, Colombia. Iran. J. Public Health.

[14] Roberts H.C., Denison H.J., Martin H.J., Patel H.P., Syddall H.E., Cooper C., Sayer A.A. (2011). A review of the measurement of grip strength in clinical and epidemiological studies: Towards a standardised approach. Age Ageing.

[15] Alahmari K.A., Silvian S.P., Reddy R.S., Kakaraparthi V.N., Ahmad I., Alam M.M. (2017). Hand grip strength determination for healthy males in Saudi Arabia: A study of the relationship with age, body mass index, hand length and forearm circumference using a hand-held dynamometer. J. Int. Med. Res..

[16] Al Risi A.-M., Al Busaidi S., Al Aufi H., Al Hashmi L., Sirasanagandla S.R., Das S. (2025). Anatomical study of the palmaris longus muscle and its clinical importance. Diagnostics.

[17] Fan S., Cepek J., Symonette C., Ross D., Chinchalkar S., Grant A. (2019). Variation of grip strength and wrist range of motion with forearm rotation in healthy young volunteers aged 23 to 30. J. Hand Microsurg..

[18] Le Roux H. (2025). Wrist Flexion and Extension: A Guide to Measuring, Testing, and Improving Range of Motion and Strength. BTE Technologies. https://www.btetechnologies.com/therapyspark/wrist-flexion-and-extension/.

[19] Asadujjaman M., Molla M.B.A., Noman S.N.A. (2019). Stature estimation from hand anthropometric measurements in Bangladeshi population. J. Forensic Leg. Med..

[20] Porretto-Loehrke A., Schuh C., Szekeres M. (2016). Clinical manual assessment of the wrist. J. Hand Ther..

[21] Kim T.S., Park D.D.H., Lee Y.B., Han D.G., Shim J.S., Lee Y.J., Kim P.C.W. (2014). A study on the measurement of wrist motion range using the iPhone 4 gyroscope application. Ann. Plast. Surg..

[22] Chen Y.-L., Chiu P.-Y. (2025). Age and sex differences in wrist range of motion: A population-based study of young and elderly Taiwanese adults. Int. J. Ind. Ergon..

[23] Dodds R.M., Syddall H.E., Cooper R., Benzeval M., Deary I.J., Dennison E.M., Der G., Gale C.R., Inskip H.M., Jagger C. (2014). Grip strength across the life course: Normative data from twelve British studies. PLoS ONE.

[24] Popp W.L., Richner L., Lambercy O., Shirota C., Barry A., Gassert R., Kamper D.G. (2023). Effects of wrist posture and stabilization on precision grip force production and muscle activation patterns. J. Neurophysiol..

[25] Massy-Westropp N.M., Gill T.K., Taylor A.W., Bohannon R.W., Hill C.L. (2011). Hand grip strength: Age and gender stratified normative data in a population-based study. BMC Res. Notes.

[26] Bhardwaj P., Nayak S.S., Kiswar A.M., Sabapathy S.R. (2011). Effect of static wrist position on grip strength. Indian J. Plast. Surg..

[27] Napper A.D., Sayal M.K., Holmes M.W.R., Cudlip A.C. (2023). Sex differences in wrist strength: A systematic review. PeerJ.

[28] Chaparro A., Rogers M., Fernandez J., Bohan M., Choi S.D., Stumpfhauser L. (2000). Range of motion of the wrist: Implications for designing computer input devices for the elderly. Disabil. Rehabil..

[29] Puh U. (2010). Age-related and sex-related differences in hand and pinch grip strength in adults. Int. J. Rehabil. Res..

[30] Fallahi A.A., Jadidian A.A. (2011). The effect of hand dimensions, hand shape and some anthropometric characteristics on handgrip strength in male grip athletes and non-athletes. J. Hum. Kinet..

[31] Lee M.-R., Jung S.M., Bang H., Kim H.S., Kim Y.B. (2018). Association between muscle strength and type 2 diabetes mellitus in adults in Korea: Data from the Korea National Health and Nutrition Examination Survey (KNHANES) VI. Medicine.

[32] Bohannon R.W., Smith J., Barnhard R. (1994). Grip strength in end stage renal disease. Percept. Mot. Ski..

[33] Peterson M.D., Duchowny K., Meng Q., Wang Y., Chen X., Zhao Y. (2017). Low normalized grip strength is a biomarker for cardiometabolic disease and physical disabilities among US and Chinese adults. J. Gerontol. A Biol. Sci. Med. Sci..

[34] Hu S., Gu Y., Lu Z., Zhang Q., Liu L., Meng G., Yao Z., Wu H., Bao X., Chi V.T.Q. (2019). come before Zhang QRelationship between grip strength and prediabetes in a large-scale adult population. Am. J. Prev. Med..

[35] Cheung C.-L., Nguyen U.-S.D.T., Au E., Tan K.C.B., Kung A.W.C. (2013). Association of handgrip strength with chronic diseases and multimorbidity. Age.

[36] Peters P.G., Alessio H.M., Hagerman A.E., Ashton T., Nagy S., Wiley R.L. (2006). Short-term isometric exercise reduces systolic blood pressure in hypertensive adults: Possible role of reactive oxygen species. Int. J. Cardiol..

[37] Stahl S., Stahl A.S., Meisner C., Hentschel P.J.H., Valina S., Luz O., Schaller H.E., Lotter O. (2013). Critical analysis of causality between negative ulnar variance and Kienböck disease. Plast. Reconstr. Surg..

[38] Eiken O., Niechajev I. (1980). Radius shortening in malacia of the lunate. Scand. J. Plast. Reconstr. Surg..

[39] Cha S.M., Shin H.D., Kim K.C. (2012). Positive or negative ulnar variance after ulnar shortening for ulnar impaction syndrome: A retrospective study. Clin. Orthop. Surg..

[40] Boulas H.J., Milek M.A. (1990). Ulnar shortening for tears of the triangular fibrocartilaginous complex. J. Hand Surg. Am..

[41] Tomaino M.M., Elfar J. (2005). Ulnar impaction syndrome. Hand Clin..

[42] Kitsoulis P., Paraskevas G., Iliou K., Kanavaros P., Marini A. (2010). Clinical study of the factors affecting radioulnar deviation of the wrist joint. BMC Musculoskelet. Disord..

[43] Aranceta-Garza A., Ross K. (2021). A comparative study of efficacy and functionality of ten commercially available wrist-hand orthoses in healthy females: Wrist range of motion and grip strength analysis. Front. Rehabil. Sci..

